# Prevalence, socio-demographic determinants, and self-reported reasons for hysterectomy and choice of hospitalization in India

**DOI:** 10.1186/s12905-022-02072-7

**Published:** 2022-12-12

**Authors:** Priyanka Kumari, Jhumki Kundu

**Affiliations:** International Institute for Population Sciences, Mumbai, 400088 India

**Keywords:** Hysterectomy, Prevalence, Determinants, Public, Private, India

## Abstract

**Background:**

There is limited evidence of hysterectomy in India because of a lack of data in large-scale, nationally representative health surveys. In 2015–16, the fourth National Family Health Survey (NFHS-4)—a cross-sectional survey—collected for the first-time direct information on hysterectomy and self-reported reasons for undergoing the procedure among women in the reproductive age group. The current study evaluates the prevalence, determinants, and choice of hospitalization (Public vs. Private) for conducting hysterectomy in India among women aged 15–49 years in 29 states and seven union territories (UTs) based on the new large-scale population-based nationally representative dataset (NFHS 5).

**Methods:**

Cross-tabulations and percentage distributions were utilized to analyse the prevalence of hysterectomy and the choice of hospitalization (public vs. private) across different socioeconomic backgrounds and reasons for undergoing hysterectomy. A multivariate binary logistic regression model was also used to find statistically significant determinants of hysterectomy.

**Results:**

In India as a whole, 3.3% of women aged 15–49 years had undergone a hysterectomy. The percentage of women who had undergone the procedure was found to vary considerably across the states and the UTs. The southern region stands out for the considerably higher prevalence of hysterectomy; particularly in the states of Andhra Pradesh (8.7%) and Telangana (8.2%), the prevalence was very high followed by Bihar (6%) and Gujrat (4%). On the other hand, the North-eastern region had the lowest prevalence of hysterectomy (1.2%). A noticeable fact that emerged was that the majority of the hysterectomies were performed in the private sector (69.6%) in India. But the scenario was quite different in north-eastern region as in this region more hysterectomies were performed in public health facilities (73%) rather than private health facilities (26.7%). Age, place of residence, religion, caste, level of education, geographic region, wealth quintiles, parity, age at first cohabitation of women were found to be the socio-demographic determinants statistically associated with hysterectomy in India. The likelihood of hysterectomy was higher among women living in rural areas (AOR: 1.3, CI: 1.23–1.35), in the richest wealth quintile (AOR 2.6; CI 2.37–2.76) and in the southern region (AOR 1.6; CI 1.47–1.66). The reasons frequently reported for hysterectomy were excessive menstrual bleeding/pain, followed by fibroids/cysts.

**Conclusion:**

This study has attempted to analyse hysterectomy prevalence and its socio-economic determinants using the latest fifth round of NFHS data of all the states and covering 21 states and union territories of India, which gives wider coverage of hysterectomy and more recent with accurate data. More research is needed therefore to unravel the complex dynamics of hysterectomy in India (and elsewhere) which could be used to help women make more informed choices and in turn advance their reproductive health and rights.

## Background

A hysterectomy is a surgical procedure in which a woman's uterus is removed. There are several varieties of hysterectomy, including partial, complete, and radical. In many parts of the world, hysterectomy, or the surgical removal of the uterus, is the second most common non-obstetric surgery after caesarean section [1–4]. Furthermore, prophylactic oophorectomy, which involves the removal of the ovaries, is sometimes suggested in conjunction with hysterectomy to lower the risk of ovarian cancer in the future [5].

Gynecological conditions such as fibroids, dysfunctional uterine hemorrhage, and uterine prolapse are common medical reasons for hysterectomy [6]. The surgical removal of a woman's uterus and ovaries can have major physical and psychological implications. According to research, there are both positive and negative consequences. On the one hand, hysterectomy has been shown to reduce anxiety and depression in women and thereby enhance their quality of life, particularly 6 to 12 months after surgery, by alleviating gynecological disorders such as irregular bleeding and pelvic pain [7–9].

Due to differences in uterine pathology, provider and patient characteristics, and socio-cultural factors, the frequency and prevalence of hysterectomy vary substantially across different geographic locations [3, 10, 11]. Because most hysterectomy research is conducted on inpatient hospitals and community-based studies, sample demographics and techniques might make worldwide comparisons of hysterectomy rates difficult. Nonetheless, research reveals that hysterectomy rates in developed countries are substantially greater than in low-income countries [10]. Hysterectomy rates are declining in many regions of the developed world, according to new research, as less invasive alternatives to hysterectomy, including as endometrial ablation and uterine artery embolization, become more commonly available. Hysterectomy rates have fallen in recent years in the United States and Canada, for example [2, 3]. Hysterectomy, on the other hand, appears to be on the rise in some developing countries [12, 13].

In recent years, hysterectomy has garnered more attention in India's health policy debates. A series of media reports have highlighted an unexpected jump in the number of women receiving hysterectomy in several parts of the country, with a considerable proportion of instances involving young and pre-menopausal women from poor households as the catalyst for heightened attention [14–16]. According to a study by Kameswari and Vinjamuri (2013), 60 percent of hysterectomies were performed on women under 30 in Andhra Pradesh between 2008 and 2010, and 95 percent of the operations were performed in private hospitals; the hospital discharge summaries for these operations were mostly blank, with no information regarding the procedure or follow-up instructions [17].

In many countries, including India, a number of research have looked at the socioeconomic, demographic, and residence-related factors of hysterectomy [18–21]. The risk factors for peripartum hysterectomy were studied in a cohort research. The study showed that placenta praevia/accreta is linked to a higher incidence of peripartum hysterectomy, based on data from 193 hospitals in 21 countries across Africa, Asia, Europe, and the Americas. Asian women had a greater rate of hysterectomy (7%) than African women (5%). The study also discovered that advanced maternal age, caesarean section, and giving numerous births in Asia are all risk factors [18].

Hysterectomy was more common in women over the age of 35, according to a study conducted in three villages in Haryana's Panchkula district. The most common reason for hysterectomy was excessive monthly bleeding (52/70; 74 percent); other reasons were uterine prolapse and fibroids [22]. Uikey, Wankhede, and Tajne (2018) discovered that fibroid uterus (65.33 percent) was the most common reason for hysterectomy in Maharashtra state of India. They concluded that in a developing nation like India with limited healthcare resources, non-descent vaginal hysterectomy outperforms abdominal and laparoscopic aided vaginal hysterectomy and should be the treatment of choice for benign uterine diseases [23].

In India, knowledge on hysterectomies is limited, in part due to a paucity of data from large-scale national representative surveys. As per a study based on fourth national family health survey (NFHS 4) by Singh and Govil 2021 [24], the prevalence of hysterectomy operations in India was 3.2% among women aged 15–49 years. Women with poor income, those who are older, rural women, married women, and women with more surviving children were all found to be at a higher risk for hysterectomy in two mixed method studies conducted in Gujarat, India. The average age of hysterectomy was 36 years, and the majority of the women had their hysterectomies at private health institutions, according to this study [10, 19]. Some researchers and activists have raised concerns about unnecessary hysterectomies being performed in some parts of India for commercial reasons rather than medical necessity, especially at a considerably younger age in places such as Andhra Pradesh [25–27]. There has also been a lot of debate concerning the effectiveness of elective hysterectomy, because women's reproductive health difficulties don't stop there [28]. Many health concerns arise after a hysterectomy, including: i) early menopause, ii) increased risk of cardiovascular disease, iii) increased risk of stroke, iv) urinary incontinence, v) loss of sexual desire, and vi) other health problems [10, 19].

The majority of the literature on hysterectomies comes from research conducted in developed countries or clinic samples. The scope and nature of the literature accessible about India are restricted. The limited evidence on hysterectomy in India comes from the community studies, and to our knowledge, no large-scale nationally representative dataset has been used to undertake a population-based study that can encompass India as a whole. In India, nationally representative reliable statistics are rarely available on this important aspect of women’s health.

In 2015–16, the fourth National Family Health Survey (NFHS-4)—a cross-sectional survey—collected for the first-time direct information on hysterectomy and self-reported reasons for undergoing the procedure among women in the reproductive age group. There are few studies on hysterectomy based on NFHS-4, 2015–16 [24, 29, 30]. More research is needed to understand the current situation of the prevalence of hysterectomy, its associated causes, and reasons for conducting hysterectomy because hysterectomy has such long-term impacts on a woman's health and longevity. Having noted the gaps in the previous literature on hysterectomy in India and the availability of a new large-scale population-based nationally representative dataset (NFHS 5) the current study explored the prevalence and predictors of hysterectomy in women aged 15–49 years in India.

The following questions are addressed in this paper:(i) to determine the national, state, UT, and regional prevalence of hysterectomy among women aged 15–49 years in India, (ii) to examine the socio-demographic determinants of hysterectomy, and (iii) to investigate the reasons reported by women for hysterectomy (iv)To assess the choice of hospitalization (Public vs Private) for conducting hysterectomy.

## Methods

The data used this study came from the fifth round of the National Family Health Survey (NFHS-5), which took place between 2019 and 21 under the stewardship of the Ministry of Health and Family Welfare (MoHFW), Government of India, and was coordinated by the International Institute of Population Sciences (IIPS), Mumbai. The National Family Health Survey (NFHS) is a multi-round, large-scale survey conducted in a nationally representative sample of households. The survey collected data on infant and child mortality, fertility, reproductive health, maternal and child health, nutrition, anaemia, and family planning services at the national and state levels in India. Each successive round of the NFHS has two specific goals. One is to provide required data on health and family welfare needed by the Ministry of Health and Family Welfare and other agencies for policy and program purposes, and the other is to give information on important emerging health and family welfare issues.

**Fig. 1:**
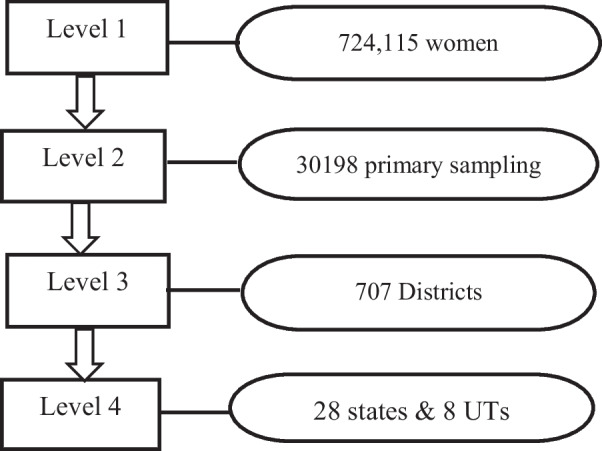
Design of the study

The NFHS-5 was based on a stratified two-stage sampling design that yielded state representative samples after applying sampling weights to control the complex survey design. The data had four levels of hierarchical structure with individuals at level 1, PSUs at level 2, districts at level 3 and state/union territories at level 4 (Fig. 1). This survey collected information from a nationally representative sample of 636,699 households, with 724,115 women aged 15–49 years and 101,839 men aged 15–54 years, with an overall response rate of 98 percent. All participants provided informed consent to sign to participate and to allow their data to be used for research. In this analysis, we included all the women of age 15–49.

## Outcome and independent variables

Hysterectomy was utilized as the outcome variable in this study. The NFHS-5 posed a series of questions to women about hysterectomy. The first question asked was: "*When did your last menstrual period start?*" (*Question no 250 of NFHS 5 women’s questionnaire*) [31]. Among the several answers to this question, one of the options was "*Has had a hysterectomy*". The direct question on hysterectomy canvassed was, "*Some women undergo an operation to remove the uterus. Have you undergone such an operation?*" (*Question no 253 of NFHS 5 women’s questionnaire*) [31]. If the answer was yes, women were asked further questions about the timing and place of and the reason for the hysterectomy. Table 1 lists the independent variables, their category, and definitions.

**Table 1:** Independent variables, their categorization and their definition

Independent variable	Definition	Categories
Age	Biological age of women respondent	15–29; 30–39; 40–49
Residence	Place where the respondent usually Lives	Urban; Rural
Religion	Religion in which the respondent believes	Hindu; Muslim (Islam); Christian; Others (Sikh; Buddhist/neo-Buddhist; Jain, Jewish, parsi/zorostrian, no religion & others)
Caste/Tribe	Scheduled Caste/Tribe, Other Backward Classes as defined in the Indian constitution for the socially and economically deprived sections of the Society	Scheduled caste; Scheduled tribe; Other Backward Class; Others (does not belong to any of the above three groups)
Women’s education	Educational attainment of women depending on years of schooling	No Schooling (0 years of schooling); Primary complete (5 years of schooling); Secondary complete (6–12 years of schooling); Higher (13 years and above schooling)
Wealth Index	Household wealth index created by using scores of possessions of certain goods and assets and classified in quintiles. Score moving from lowest to highest means household moving from poor to rich category	Lowest; Second; Middle; Fourth; Highest
Marital Status	Current marital status of women; Others includes divorce, separated, and living together without marriage	Currently Married; Widow; Others (Never married, divorced, separated)
Parity	Total no. of children ever born to Women	0; 1 child; 2 children; 3 and above Children
Age at first cohabitation	Age (in years) at which woman started living with spouse	Less than 15; 15–19; 20 and above
Region	Region comprising a group of states, depending upon the geographical region and the sociocultural milieu these states fall in North (Chandigarh, Delhi, Haryana, Himachal Pradesh, Jammu & Kashmir, Punjab, Rajasthan, Uttarakhand); Central (Chhattisgarh, Madhya Pradesh, and Uttar Pradesh); East (Bihar, Jharkhand, Odisha, West Bengal); Northeast (Arunachal Pradesh; Assam, Manipur, Meghalaya, Mizoram, Nagaland, Sikkim, Tripura); West (Dadra & Nagar Haveli, Daman & Diu, Goa, Gujarat, Maharashtra); South (Andaman & Nicobar Island, Andhra Pradesh, Karnataka, Kerala, Lakshadweep, Puducherry, Tamil Nadu, Telangana)	North; Central; East; Northeast; West; South

STATA 16 was used to conduct univariate, bivariate, and multivariate analyses for this study. Univariate analyses were used to estimate the prevalence of hysterectomy. Bivariate analyses were performed to determine the prevalence of hysterectomy in various states and regions of India and to determine the unadjusted associations between the selected socio-economic, demographic, and biological factors with hysterectomy. Finally, multivariate analyses using binary logistic regression were conducted to determine the relations of various factors to the dependent variable, hysterectomy.

The dependent variable was dichotomous with mutually exclusive categories, i.e., had undergone a hysterectomy or had not undergone a hysterectomy. The independent variables were categorical; thus, performing binary logistic regression was the most appropriate approach. The parameters in the logistic regression models were estimated using the maximum likelihood method, and the model's goodness of fit was determined using pseudo-R2 statistics. Results are presented in the form of odds ratios (ORs) with 95% confidence intervals (CI). The analyses were conducted using appropriate sampling weights. A thematic map was also created using a geographic information system (GIS).

## Results

### Prevalence and regional distribution of hysterectomy in India

According to the most recent NFHS-5 empirical data, the percentage of women who have had a hysterectomy in India is not low. The percentage of women who have undergone a hysterectomy was 3% among women aged 15–49 (Table 2). Table 2 also provides regional variations in the level of hysterectomy in India. The prevalence of hysterectomy was highest in Southern region, i.e., 4.2%, which was also greater than the national prevalence, followed by Eastern part of India (3.8%). On the other hand, the lowest prevalence was observed in the Northeast region, i.e., only 1.2%.

**Table 2:** Percentage of women age 15–49 who have had a hysterectomy, and among women with a hysterectomy according to background characteristics, India, 2019–21

Background characteristics	Number of women	Number of women with hysterectomy	Percentage
*Age*			
15–29	359,152	804	0.2
30–39	197,936	6564	3.3
40–49	167,050	16,247	9.7
*Place of residence*			
Urban	235,278	5988	2.3
Rural	488,836	17,627	3.6
*Religion*			
Hindu	589,164	20,233	3.4
Muslim	97,595	2247	2.3
Christian	16,995	553	3.3
Others (Sikh, Buddhist/neo-buddhist, jain, jewish, parsi/Zoroastrian, no religion & other)	20,360	581	2.9
*Caste*			
Schedule caste (SC)	158,482	4911	3.1
Schedule tribe (ST)	67,262	1459	2.1
Other backward class (OBC)	310,782	11,284	3.6
Others	187,586	5960	3.2
*Education level*			
No education	162,450	11,618	7.2
Primary	84,922	3949	4.7
Secondary	363,395	7206	2.0
Higher	113,346	842	0.7
*Wealth Index*			
Poorest	133,973	3820	2.9
Poorer	144,813	5049	3.5
Middle	148,616	5483	3.7
Richer	150,680	5205	3.5
Richest	146,032	4056	2.8
*Marital status*			
Currently married	521,352	21,624	4.2
Widowed	22,597	1677	7.4
Others (Never married/divorced/separated)	180,165	314	0.2
*Parity*			
No children	223,105	435	0.2
1 child	103,185	1732	1.7
2 children	195,458	8117	4.2
3 & above	202,365	13,332	6.6
*Age at first cohabitation#*			
< 15 years	65,272	5569	8.5
15–19 years	340,003	14,703	4.3
20 & above years	146,764	3279	2.2
*Region*			
North	102,199	2322	2.3
Central	180,228	4573	2.5
East	164,828	6269	3.8
North-east	26,745	319	1.2
West	71,849	2591	3.6
South	178,623	7541	4.2
Total	724,115	23,616	3.3

^#^The ‘N’ is not additive to the total ‘N’ mainly because of flagged and missing cases

### Socio-economic differentials in hysterectomy in India

Table 2 depicts the percentage of women aged 15–49 who have had a hysterectomy by socio-economic and demographic characteristics. A considerable variation in women's socio-economic and demographic characteristics was observed in the risk of hysterectomy. The level of hysterectomy increased with an increase in age. A smaller percentage of women (0.2%) aged 15–29 reported having undergone a hysterectomy; this percentage increased to 3.3% among women aged 30–39, and 9.7% among women aged 40–49. Rural women (3.6%) were at a higher risk of hysterectomy than urban women (2.3%). The percentage of hysterectomized women was highest (3.4%) among Hindus, Christians, and other religious groups and lowest among Muslims (2.3%).

Women from other backward classes were found to have a higher percentage (3.6%) of hysterectomy than women from scheduled tribes (2.1%). Women with a higher education had a lower percentage of hysterectomy (0.7%) than women with no education (7.2%). As a result, hysterectomy was performed on 4.7% of women aged 15–49 with a "primary complete" level of education and 2% of women with a "secondary complete" level of education. There was no substantial difference in hysterectomy rates among women from different wealth quintiles.

Table 2 clearly reveals that widows had a higher proportion (7.4%) of hysterectomy than women in the others category (0.2%), followed by currently married women (4.2%).

In terms of parity, the percentage of hysterectomized women grew as a woman's parity increased, peaking at 6.6% among women with a third or higher order parity. The percentage of hysterectomized women was found to be high (8.5%) among women who began cohabitation at a younger age, notably under the age of 15.

Table 2 also provides regional variations in the level of hysterectomy in India. The South, west, and east (4%) regions showed a prevalence of hysterectomy above the national level (3%). The percentage of hysterectomy (2.3%) for the North regions turns out to be slightly lower than the national average (3%). In contrast, the least proportion of women undergoing hysterectomy was found in the North-east region (1.2%).

**Fig. 2:**
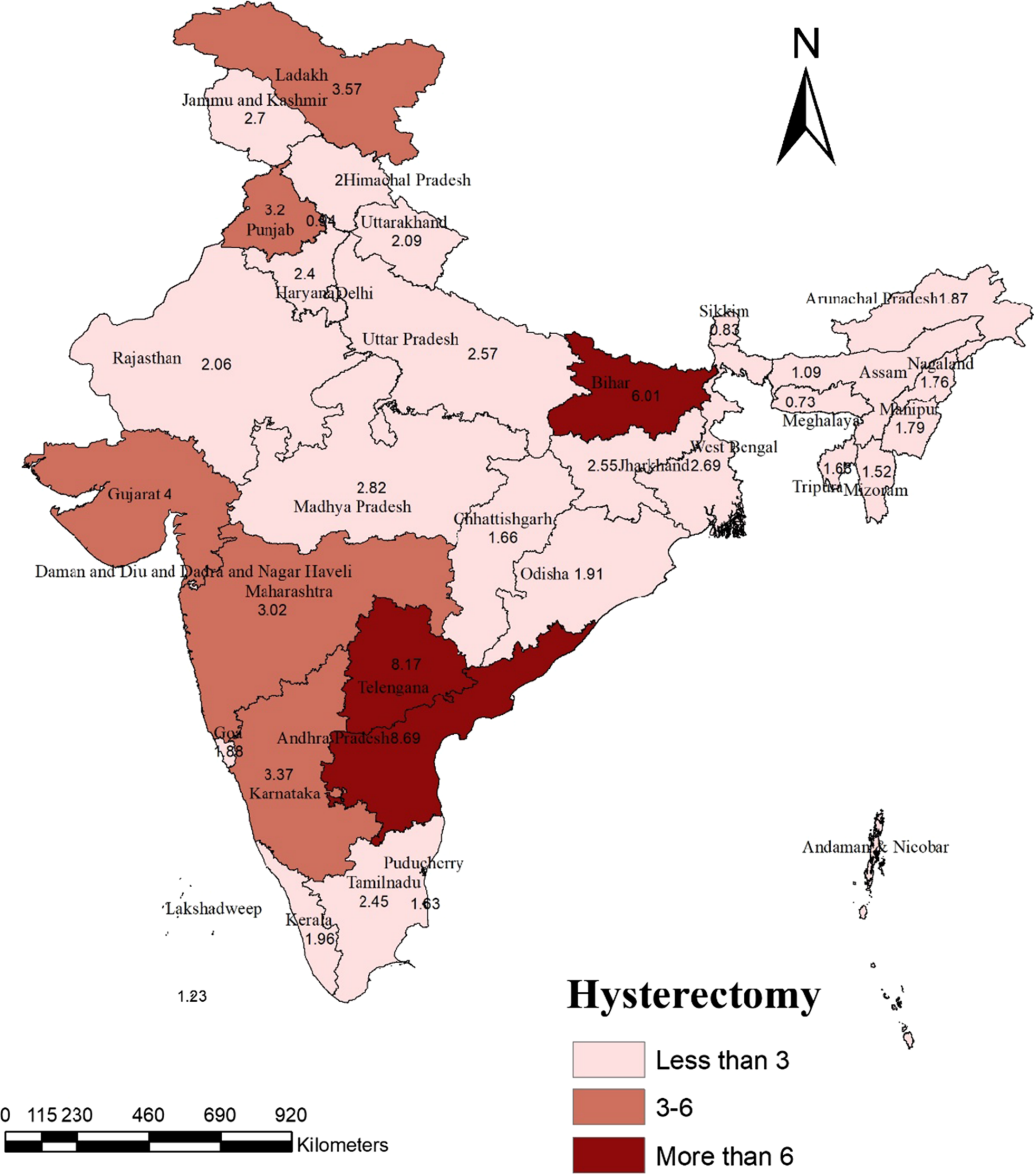
Prevalence of hysterectomy by states/UTs, India, NFHS 5, 2019–2021

In India, the proportion of women aged 15–49 who get a hysterectomy varies significantly by geographical region. Figure 2 reveals that Among all the states, Andhra Pradesh had the highest prevalence (8.7%) of hysterectomy, followed by Telangana (8.1%) Bihar (6%) and Gujarat (4%), Whereas the lowest prevalence of hysterectomy was observed in Meghalaya with a prevalence rate of 0.7%, followed by Sikkim (0.8%) and Chandigarh. Among UTs, Lakshadweep had the lowest prevalence of hysterectomy (1.2%), followed by Puducherry (1.6%). States which had a prevalence rate above the national average were Andhra Pradesh, Telangana, Gujarat, Bihar and Ladakh.

### Contrasts in the median age of hysterectomy in India

**Fig. 3:**
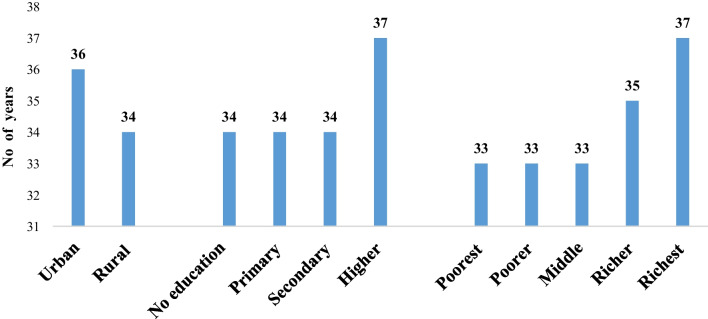
Socioeconomic contrasts in median age of hysterectomy in India, NFHS 5 (2019–21)

Figure 3 shows women's median age (in years) at hysterectomy in India and variations across the residence, education, and wealth quintiles. The median age at hysterectomy was about 2 years higher among urban women (36 years) than rural women (34 years). Women with no education had their hysterectomy at a younger median age (34 years) than women with higher education (37 years).

The median age of hysterectomy for women in the poorest wealth quintile was 4 years younger than for women in the richest wealth quintile, indicating a considerable distinction between the two groups of women. All the median ages for hysterectomy across the residence, educational level, and household wealth quintile categories, shown in Fig. 2.

### Results from multivariate logistic regression analysis

Table 3 illustrates the adjusted odds ratio (AOR) from a multivariate logistic regression that was used to look at the likelihood of a woman having a hysterectomy (dependent variable) belonging to given socio-demographic background characteristics (independent variables). A woman's age was found to be statistically associated with an increase in the risk for hysterectomy. For example, women aged 30–39 were 17.8 times more likely than women aged 15–29 to have undergone a hysterectomy. Likewise, women aged 40–49 were 7.9 times more likely than women aged 15–29 to have undergone a hysterectomy. Women in rural areas were 1.3 times more likely to have undergone a hysterectomy than women in urban areas. Muslim (AOR: 0.7, 95% CI [0.70–0.79]) and Christian (AOR: 0.8, 95%CI [0.76–0.92]) women were less likely to have had a hysterectomy compared with Hindu women. On the other hand, women who belonged to other religious groups (AOR: 1.1, 95%CI [1.01–1.21]) were more likely to have undergone a hysterectomy than Hindu women. Hysterectomy was also found to be linked to caste. Women from scheduled tribes were less likely (AOR: 0.7, 95% CI [0.68–0.77]) to have had a hysterectomy than women from scheduled castes.

**Table 3:** Odds ratios of the relation of background variables to hysterectomy: Adjusted results from logistic regression analysis, NFHS-5

Background characteristics	Adjusted Odds ratio (AOR)	95% CI
*Age*		
15–29^®^		
30–39	17.8***	16.3–19.4
40–49	7.9***	7.33–8.65
*Place of residence*		
Urban^®^		
Rural	1.3***	1.23–1.35
*Religion*		
Hindu^®^		
Muslim	0.7***	0.70–0.79
Christian	0.8***	0.76–0.92
Others (Sikh, Buddhist/neo-buddhist, jain, jewish, parsi/Zoroastrian, no religion & other)	1.1*	1.01–1.21
*Caste*		
Schedule caste (SC)^®^		
Schedule tribe (ST)	0.7***	0.68–0.77
Other backward class (OBC)	1.2***	1.12–1.23
Others	1.1***	1.05–1.17
*Level of education*		
No education^®^		
Primary	0.9***	0.86–0.95
Secondary	0.8***	0.76–0.83
Higher	0.4***	0.35–0.42
*Wealth index*		
Poorest^®^		
Poorer	1.5***	1.45–1.62
Middle	1.8***	1.67–1.87
Richer	2.1***	2.00–2.27
Richest	2.6***	2.37–2.76
*Marital status*		
Currently married^®^		
Widowed	0.8***	0.78–0.88
Others (Never married/divorced/separated)	0.2***	0.19–0.24
*Parity*		
No children^®^		
1 child	1.9***	1.71–2.19
2 children	3.4***	3.05–3.82
3 & above	2.9***	2.55–3.18
*Age at first cohabitation*		
< 15 years^®^		
15–20 years	0.7***	0.63–0.69
> 20 years	0.4***	0.38–0.42
*Region*		
North^®^		
Central	0.9**	0.85–0.96
East	1.4***	1.30–1.48
North-east	0.5***	0.47–0.56
West	1.2***	1.14–1.31
South	1.6***	1.47–1.66

^®^represents the reference category

***represents 1% level of significance, ** represents 5% level of significance and * represents 10% level of significance

Women from other backward classes (AOR: 1.1, 95% CI [1.12–1.23]) and other caste categories, on the other hand, were more likely than their scheduled caste counterparts to have undergone the procedure. Woman's education was negatively associated with hysterectomy. Compared to women with no education, those with more years of schooling were less likely (AOR: 0.9, 95% CI [0.86–0.95]) to have had a hysterectomy. Those with higher education, for example, were 0.4 times (AOR: 0.4, 95% CI [0.35–0.42) less likely to have a hysterectomy than women with no education.

Women from the richest wealth quintile had a much higher likelihood of hysterectomy than women from the poorest wealth quintile. Women in the richest quintile, for example, were 2.6 (AOR: 2.5, 95% CI [2.37–2.76]) times more likely than women in the poorest quintile to have had a hysterectomy. Similarly, compared to women in the poorest quintile, women in the poorer, middle, and richer quintiles had a significantly higher risk of hysterectomy.

The findings demonstrated that marital status was negatively associated with hysterectomy. Widow women were 0.8 times (AOR: 0.8, 95% CI [0.73–0.82]) and women who belonged to other marital status (never married, divorced, and separated) were 0.8 (AOR: 0.8, 95% CI [0.65–0.87]) times less likely to have had a hysterectomy than currently married women.

Women's parity was also found to be a major predictor of hysterectomy in our study. The study discovered that women with third and higher parities were 2.9 times (AOR: 2.9, 95% CI [2.55–3.18]) more likely than nulliparous women to have had a hysterectomy. The odds of having had a hysterectomy were 3.4 (AOR: 3.4, 95% CI [3.05–3.82]) times higher for women with second parity and 1.9 times higher for women with first parity than nulliparous women.

In the study population, age at first cohabitation (also known as age at consummation of marriage) indicated a negative and significant association with having undergone a hysterectomy. Women who had their first cohabitation between the ages of 15 and 20 had a 70% (AOR: 3.4, 95% CI [3.05–3.82]) reduced likelihood of getting hysterectomy and women who had their first cohabitation at the age of 20 or older had a 40% lower chance (AOR: 0.4, 95% CI [0.38–0.42]) of undergoing hysterectomy than women who had their first cohabitation at the age of 15 or younger.

Women in the south, west, and east of India were 1.6 (AOR: 1.6, 95% CI [1.47–1.66]), 1.2 (AOR: 1.2, 95% CI [1.14–1.31]), and 1.4(AOR: 1.4, 95% CI [1.30–1.48]) times more likely to have had a hysterectomy than women in the north. Women in the Central region, on the other hand, were around 0.9 times less likely than those in the North to have had a hysterectomy. Women in the Northeast region were about 50% less likely to report having undergone a hysterectomy than women from the North region.

### Reasons for which hysterectomy was performed

The literature suggests that hysterectomy treats several conditions and diseases. These include chronic pain, excessive bleeding, endometriosis, pelvic floor prolapses, uterine and cervical cancers, uterine disorders, etc. The NFHS-5 posed the following question to all women who had undergone a hysterectomy: "Why was this operation (hysterectomy) performed?" [31]. It was a multiple response category questions as there may be more than one reason for resorting to hysterectomy.

Table 4 is generated by tabulating these responses from the dataset. According to Table 4, the most common reason for hysterectomy at the national level was excessive menstrual bleeding/pain (52%), followed by fibroid/cyst (25%), and uterine disease (11.1%).

**Table 4:** Reasons (percentages) for hysterectomy in India, NFHS-5(2019–21)

Reasons	Percentage (%)	Number (%)
Excessive menstrual bleeding/pain	51.8	12,233
Fibroids/cysts	25.0	5891
Uterine disorder (rupture)	11.1	2616
Cancer	4.3	1005
Uterine prolapse	7.1	1687
Severe post-partum haemorrhage	3.2	765
Cervical discharge	7.0	1647
Others	7.6	1783

### Sources of hysterectomy by socio-economic characteristics of the women in India, 2019–21

Women who had hysterectomies were further asked, “Where was this operation performed?” [28]. Out of all hysterectomies performed in India, more than two-thirds (69.6%) were performed in private health-care centers, whereas only 30% were performed in public health-care centers (Table 5).

**Table 5:** Percent distribution of women who had hysterectomy by place the hysterectomy was performed, according to background characteristics, India, 2019–21

Background characteristics	Public	Private
*Age*		
15–29	35.4	64.6
30–39	25	75
40–49	32	68
*Place of residence*		
Urban	31	68.7
Rural	30	70
*Religion*		
Hindu	30	70
Muslim	32.5	67.5
Christian	27.3	72.7
Others (Sikh, Buddhist/neo-buddhist, jain, jewish, parsi/Zoroastrian, no religion & other)	40.8	59.2
*Caste*		
Schedule caste (SC)	35	65
Schedule tribe (ST)	42.9	57.1
Other backward class (OBC)	25	75
Others	31.8	68.2
*Education*		
No education	30.5	69.5
Primary	33.7	66.3
Secondary	29.4	70.6
Higher	18.6	81.4
*Wealth index*		
Poorest	35	65
Poorer	34	66
Middle	30	70
Richer	28.5	71.5
Richest	23.9	76.1
*Marital status*		
Currently married	29.8	70.2
Widowed	37	63
Others (Never married/divorced/separated)	32.5	67.5
*Parity*		
No children	43.8	56.2
1 child	36	64
2 children	29.3	70.7
3 & above	29.9	70.1
*Age at first cohabitation*		
< 15 years	29.9	70.1
15–20 years	29.4	70.6
> 20 years	35.7	64.3
*Region*		
North	42.9	57.1
Central	30.9	69.1
East	27.6	72.4
North-east	73	26.7
West	41.3	58.7
South	23.0	77
Total	30.4	69.6

It's worth noting that non-governmental organizations (NGOs) and not-for-profit trusts make up a relatively small percentage of private health facilities (almost 1%). Surprisingly, 70% of women in the rural area chose private health care for the hysterectomy, which was greater than the urban area (69%). The Hindu (70%) and the Christian (73%) women were also opted for a private health care facility for hysterectomy.

65% of women of the schedule caste chose private health care facility for hysterectomy as compared to 57% of women of the scheduled tribe. 81% of women with higher education went to private health-care facilities for hysterectomy, followed by women of secondary level education (71%) and women with no education (70%). Interestingly, 65% of women from poorest wealth quintile went to private health care facility for the hysterectomy. A higher percentage of women from richest wealth quintile (72%) went to private health facilities to undertake the hysterectomy.70% of the currently married women and women with 3 or more children chose private health facilities for the hysterectomy.

The pattern in the utilization of hospitalisation (public vs private) for hysterectomy in northeast regions was quite different from the rest of the country. The utilization of the public sector was highest in the northeast region (73%), followed by the north (42.9%). 77% of hysterectomies in the southern region were done in private institutions, followed by 72% in the east.

## Discussion

The present study provides social, economic, and demographic determinants along with self-reported reasons for undergoing hysterectomy. The study also reveals the choice of hospitalization (Public vs Private) for conducting a hysterectomy. This paper comprehensively analyses all these critical aspects of hysterectomy in the Indian context. In India as a whole the percentage of women who had hysterectomies have remained same from 2015–16 (NFHS 4) to 2019–21 (NFHS-5) 3%. The findings of this study reveal that three in every 100 women aged 15–49 have had a hysterectomy in India. This study also discovered a hysterectomy prevalence in India ranging from 0.7 to 8.7 per 100 women in the age group 15–49 years, which is supported by Prusty et al.’s (2018) study, which discovered a hysterectomy prevalence ranging from 0.2 to 6.3 per 100 women in the age group 15–49 years in 21 of India's 36 states and union territories [32]. The southern region stands out for the considerably higher prevalence of hysterectomy; particularly in the states of Andhra Pradesh and Telangana, the prevalence was very high followed by Bihar and Gujrat. On the other hand, the North-eastern region had the lowest prevalence of hysterectomy. Prusty, Choithani, and Gupta also found that Andhra Pradesh (6%), Telangana (5.5%), and Karnataka (3%) had a higher prevalence than the other 18 states of India [32]. Singh et al. [24] in their study also revealed that the prevalence of hysterectomy was higher in the southern states like Andhra Pradesh and Telangana, followed by Bihar and Gujrat while the north-eastern states had the lowest prevalence.

The NFHS-5 shows that about two-thirds of women in the reproductive age group in Andhra Pradesh and about 30% in Telangana were overweight or obese [31]. The fact that hysterectomy is linked to obesity and overweight and that women in these two states confront early marriage and childbirth could explain why the prevalence of hysterectomy is higher in these two states. These higher prevalence of hysterotomies in Andhra Pradesh and Telangana also may be attributed to the State Government’s Aarogyashri health insurance scheme. Aarogyashri health insurance scheme, initiated in 2007, offers coverage for up to 1.5 lakhs (about $2 500) in medical expenses to people from low-income families, including expenses for hospitalisation and surgery at private hospitals [33]. Even though the state government implemented limits on private hospitals in 2010 [34], the association between health insurance and hysterectomy may be a potential explanation for the higher occurrence of hysterectomy in Andhra Pradesh and Telangana.

Bihar is one of the least developed states in terms of socio-economic development, and women in this state are generally unaware of reproductive health issues and treatment choices. It is also one of India's least urbanized states. The increased occurrence of hysterectomy in Bihar is likely due to low access to public health infrastructure in the state's rural areas, resulting in a delay in seeking treatment for reproductive health issues and the adoption of hysterectomy as a last resort. Gujarat, one of India's most developed states, has a large percentage of women who fall into the highest income quintile and a thriving private healthcare, both of which may contribute to an increased risk for hysterectomy [30].

According to our findings, the median age at hysterectomy for women residing in rural areas, without no education, and belonging to the poorest wealth quintile was 33–34 years. Desai et al. (2016) found similar results in research in Gujarat, where the median age of hysterectomy was 36 years for women in a low-income context [19]. Because of the long-term repercussions of having a hysterectomy at a young age, this can have a significant impact on women's socio-psychological and physical health [35].

Women with no education, those living in rural areas, those in richest wealth quintiles, those with a young age at first cohabitation, and those from the eastern, western and southern regions were more likely to have hysterectomy, according to the study. Desai, Sinha, and Mahal's study also [19] shows that rural women are more likely to have hysterectomies. Singh et al. in [36] their study also found that women from rural areas were more likely to have undergone hysterectomy than the urban women. Women in rural areas may choose to have hysterectomy because to issues such as lack of skilled gynaecologists in the village, poor cleanliness, menstrual disorders, and most importantly, taboos linked with menstruation [37]. Higher prevalence of hysterectomy in rural women is also consistent with qualitative research that reports limited non-surgical options for bleeding compared with urban women due to lack of treatment options and opportunity costs associated with pursuing non-surgical treatment–suggesting that hysterectomy may be offered as primary treatment in some rural settings [12, 38].

Women with no education and those from rural areas are more likely to have undergone a hysterectomy due to infection or uterus-related morbidities. Women from well-off households, on the other hand, may have had it since they were more likely to be able to afford the hysterectomy procedure [30].

In India, there are typical characteristics of reproduction among rural women and women without a formal education. Less-educated women are generally less informed about reproductive health and hygiene [39]. Uneducated women and those from low socio-economic origins had limited awareness of health check-ups and health-seeking behaviour. These factors may cause women to delay or avoid getting treatment in the early stages of a reproductive health problem [40]. Most women do not seek treatment for reproductive health problems in the early stages because they believe they are normal for women. In the women's reproductive health system, medical interventions are sometimes viewed as unneeded intervention [22].

The present study revealed that the leading self-reported causes of hysterectomy were excessive menstrual bleeding/pain (52%), followed by the presence of fibroids/cysts (25%) and uterine ruptures (11%) among women in 15–49 years in India. This finding is similar with the findings of Meher and Sahoo [29] Desai et al. [41], and Singh et al. [36]. However, Fibroids (73 percent in Hong Kong, 65 percent in India, 60 percent in the United States, 33 percent in Pakistan, and 23 percent in South Africa), followed by prolapse, remain the most common reasons for hysterectomy in other nations [42–45]. Therefore, specific interventions are needed to address issues with reproductive health, particularly those that were reported as causes of hysterectomy in this study.

A noticeable fact that emerged was that the majority of the hysterectomies were performed in the private sector in India. But the scenario was quite different in north-eastern region as in this region more hysterectomies were performed in public health facilities rather than private health facilities (26.7%). This result is in line with the research done by Meher and Sahoo 2020 [46] that public health care centres are still preferred by a sizable percentage of women in India's north-eastern area for hysterectomy procedures, despite the fact that private healthcare facilities are trending upward in all regions and public facilities are trending downward. This is because the North-eastern states are in a better condition as compared to other states of the country in terms of physical health care infrastructure. There has been significant improvement in the rural health care infrastructure, especially after the implementation of National Rural Health Mission [47, 48]. Another factor would be that it can be difficult to get private health care facilities in remote parts of all the North-eastern states within safe physical reach [48]. Desai et al. in their study also showed that almost two-thirds of women undergoing hysterectomy utilized private hospitals, while the remainder used government or other non-profit facilities [10].

This study had some strengths and limitations. One of the study's main advantages was the wider applicability of its findings because it was based on information from a significant health survey conducted in India. The following were the study's limitations. First, the study did not show any information on history of hysterectomy and only self-reported prevalence of hysterectomy was taken into account in this study because it used data from a large-scale survey. Second, recall biases or reporting biases may have affected the self-reported hysterectomies. Third, because this study was cross-sectional, it was impossible to determine the temporal and potentially causal relationship between the variables. Fourth, further information about hysterectomy, such as the type of hysterectomy performed, issues women face after a hysterectomy, expenses of hysterectomy, etc., was not available due to the restriction of using a secondary database. Fifth, NFHS data also did not include health insurance status at the time of hysterectomy or mode of payment, precluding an analysis of the role of health financing. Sixth, as currently reported in the NFHS, categories of self-reported causes of hysterectomy do not clearly distinguish between obstetric and non-obstetric instances and there are no data on women aged at least 50 years among whom this procedure is more common.

The present study revealed at the national level, a sizable proportion of ever-married women are undergoing hysterectomy. This may have adverse effects on the physical, socio-psychological and reproductive health of women. Thus, health education regarding gynecologic issues, probable hysterectomy side effects, and provider training come up as critical concerns. Providing more information about hysterectomy and also alternative options will enable the women to make more informed choices. To encourage women's high-quality prevention and treatment options rather than "permanent" but potentially inappropriate treatments, a rights-based approach to women's health is crucial.

## Conclusion

This study has attempted to analyse hysterectomy prevalence and its socio-economic determinants using the latest fifth round of NFHS data of all the states and union territories of India, which gives wider coverage of hysterectomy and more recent with accurate data. Although the national prevalence of hysterectomy estimate was low compared with other countries, the median age at the procedure and prevalence in specific states are of primary concern. Our findings confirm that, in order to decrease the number of women who need hysterectomies, therapy for gynaecological morbidity, such as heavy bleeding, must be given high priority. Moreover, the use of non-surgical or conservative approaches as a form of gynaecological morbidity treatment is also necessary. The study also reveals that out of all hysterectomies performed in India, more than two-thirds were performed in private health-care centers. In the private sector, there is a financial incentive for doctors to carry out procedures regardless of whether or not there is any benefit to the patient. Private clinics actively mislead women from low-income regions in India into having unneeded hysterectomies and caesarean deliveries, which entail enormous costs and hazards to their health. As a result, many women are left with debilitating debts. Women from the most discriminated low castes and poor economic backgrounds are being targeted due to the extremely limited access to free government healthcare and the high illiteracy rates.

India's health system currently caters to a limited range of health services for women, relating to pregnancy, delivery, family planning, and postpartum care. The recently introduced initiative to support Comprehensive Primary Health Care represents a great development in the fight against chronic diseases, but much more funding is needed. The management of gynaecological morbidity will require special training for primary care professionals.

## Data Availability

The data for this research is available to the public on DHS measures website. Any individual can register and easily obtained data in electronic version from the following website https://dhsprogram.com/data/new-user-registration.cfm

## Abbreviations

NFHS: National Family Health Survey
DHS: Demographic and Health Survey
OR: Odds ratio
CI: Confidence interval
